# Analysis of water quality over non-condensable gases concentration on steam used for sterilization

**DOI:** 10.1371/journal.pone.0274924

**Published:** 2022-09-27

**Authors:** Emerson Aparecido Miguel, Paulo Roberto Laranjeira, Marina Ishii, Terezinha de Jesus Andreoli Pinto

**Affiliations:** 1 Orionce Serviços de Metrologia Ltda., Barueri, SP, Brazil; 2 Cordis US Corp., Boca Raton, FL, United States of America; 3 Department of Biochemical and Pharmaceutical Technology, Faculty of Pharmaceutical Sciences, University of São Paulo, São Paulo, SP, Brazil; 4 Department of Pharmacy, Faculty of Pharmaceutical Sciences, University of São Paulo, São Paulo, SP, Brazil; Pandit Deendayal Petroleum University, India, INDIA

## Abstract

**Background:**

Non-condensable gases (NCGs) are all gases that do not undergo liquefaction during the saturated steam sterilization process. During a sterilization cycle, the NCGs presence inside the chamber is one of the biggest threats to the sterilization process compromising process validation and product quality.

**Methods:**

In this work, 170 testing of NCGs concentrations performed between September 2016 and August 2021 were carried out by Orionce Serviços de Metrologia Ltda (Barueri, SP, Brazil), according to EN285:2015 procedure. For steam generation, the types of water used were softened water (SW), one-step reverse osmosis (RO), purified water (PW), and water for injection (WFI). The data obtained were analyzed using Minitab^®^ software, version 18.1, to identify a relationship between the water quality used for steam generation and the concentration of non-condensable gases determined into equipment qualification.

**Findings:**

From total tests performed, 109 tests passed, and 61 tests failed, representing 64.2% and 35.8%, respectively. A higher failure rate was observed in terms of concentration of non-condensable gases in systems that used purified water for steam generation (64.7%), followed by softened water (55.6%), one-step reverse osmosis (42.9%), and water for injection (7.6%).

**Conclusion:**

System processes using WFI for steam generation showed better results for steam quality approvement, in terms of NCGs concentration, compared to softened, purified, or reverse osmosis water treatments in the concentration of non-condensable gases in steam used for sterilization processes of industrial utilities.

## Introduction

Steam sterilization process is widely used to process items that can withstand moisture and high temperature. Steam is water in the vapor state: it is nontoxic, generally readily available, and relatively easy to control, being used for numerous applications in the pharmaceutical, medical device industries, and hospitals [1].

To validate a sterilization process, the quality of saturated steam (97% dry steam with 3% moisture), used as a sterilizing agent, is a fundamental requirement to be proven and must be an integral part of steam sterilization process principles [2, 3]. Good quality steam can be produced by low total dissolved solids or mineral-free water such as reverse osmosis or electro deionized water [3]. Steam quality refers to the measurable physical aspect of steam used in sterilization, and it is responsible for approximately 60% of failures in sterilization processes, which compromise product quality, control of production steps, or process validation [4].

Temperature (superheat), dryness (liquid water content), and non-condensable gas (NCG) content are considered measurable critical and crucial aspects for steam used in sterilization: European standards, International Organization for Standardization (ISO) standards, and US industrial standards, including EN285, HTM 2010, and ANSI/AAMI/ISO 17665 [5–7], require these measurements as part of the qualification of a steam sterilizer equipment and during ongoing periodic validations. Deviations from established ranges of these aspects of the steam can result in wet loads or sub-optimal sterilization cycles, which are a concern because they can create a potential risk of contamination of instruments and materials after sterilization.

Non-condensable gases (NCGs) are all gases that do not undergo liquefaction during the saturated steam sterilization process, such as oxygen dissolved in water (O_2_), carbon dioxide (CO_2_), and nitrogen (N_2_) [7]. The presence of air and other non-condensable gases (NCGs) inside the chamber during a sterilization cycle is one of the biggest threats to the sterilization process. NCGs prevent the steam condensation on the medical device surface, inhibiting thermal coagulation of cell wall proteins of microorganisms, including spores, during the sterilization [5, 8–10], affecting steam quality by reducing its energy power they have a lower heat capacity compared to water. Therefore, air pockets form (bubbles of NCGs) inside the sterilizer chamber that isolate the goods to be sterilized and block further condensation of the steam before sterilization, can slow down the heating process, or the sterilization cycle may abort due to insufficient vacuum if the gases are not correctly removed [10]. The presence of NCGs constitutes a potential factor of a process failure since they occupy space in the internal chamber of the equipment, competing with water vapor and acting as a thermal insulator when the equipment is in operation. According to EN285, 2015, the volume of NCGs must not exceed 3.5% or 3.5mL per 100mL of condensate when collected according to the methodology described in the technical instruction. These gases are not detected by the equipment monitoring devices or control systems and can compromise the effectiveness of the process [4, 11].

Many studies have been developed to evaluate the effect of the NCGs in the steam generation. Feurhuber et al., 2019 [12], developed a computational fluid dynamics (CFD) model to simulate the fluid flow, temperature, heat transfer, and steam penetration inside the steam sterilizer. The CFD model developed was able to predict the volume fraction of NCGs, as well as steam penetration inside the steam sterilizer, including the hollow loads. CFD simulations can be a useful tool to investigate the various physical phenomena that occurs within steam sterilizers, as predicting steam condensation in the presence of NCGs [13], the air-removal process during the pre-sterilization phase of both vacuum and non-vacuum steam sterilization cycles and the inactivation of *Geobacillus stearothermophilus* on real dental handpieces [14].

The Brazilian National Health Surveillance Agency, ANVISA, published the Resolution of the Collegiate Board for Good Practices in the Production of Medicines, RDC 301, in August 2019, in line with Brazil’s participation in the Pharmaceutical Inspection Co-operation Scheme (PIC/S) [15]. The Good Manufacturing Practices must be assumed in standard agreement by all industry sectors as decisions can impact the result of the production chain. The process validation methodology consists of installation, operation, and performance qualification steps [16] and an establishment to evaluate the quality of the steam used in the processes [5, 17–21].

Water quality plays an essential role in the sterilization processes due to the steam generation process. Regardless of appearance, water can be a source of microorganisms or chemicals [22], and it is classified depending on the unit operations sequence performed depending on its purpose of use. For pharmaceutical industries, the water quality for steam generation is not established or defined, leading to a variation in its quality parameters and, consequently, the efficiency of final purposes.

This study aims to evaluate the relationship between the quality of water used to generate steam with the concentration of non-condensable gases determined in the qualification of the equipment, to contribute with data and discussion about the qualification of the steam quality, one requirement demanded by the Brazilian Health Regulatory Agency.

## Material and methods

### Scope of study

To assess the quality of the steam used for sterilization processes, 170 testing of non-condensable gases (NCGs) concentration determination, performed as part of validation processes between the period of September 2016 and August 2021, were carried out by the service provider in equipment qualification, Orionce Serviços de Metrologia Ltda, (Barueri, SP, Brazil). Equipment evaluated for use in distinct pharmaceutical industries areas differed in structure, complexity, and functionality, such as autoclaves, bioreactors, reactors, filling machines, and tanks.

### Water treatment methods

For a steam generation, industrial facilities evaluated used four types of water: softened water (SW), one-step reverse osmosis (RO), purified water (PW), and water for injection (WFI) characterized according to specifications of International Standards, guidelines, and pharmaceutical compendiums.

All water used for steam generation comes from potable water and follows a general scheme of production. After the filtration step to remove particles from 5 to 10 μm, water is filtrated by adsorption in an activated carbon filter, followed by chemical treatment with acid and base to remove carbonates and ammonia, and with pH adjustment to the range between 6.0 to 8.0. The softened water is carried out after these steps and passes by ion exchange columns to capture calcium and magnesium ions. A reverse osmosis step removes ions, microorganisms, bacterial endotoxins, and most contaminants without adding additives such as NaOH to remove Organic Carbon such as CO_2_. Purification can be performed by various water techniques, the most common being double-step reverse osmosis, where NaOH is added to scavenge CO_2_. Purified Water (PW) and Water For Injection (WFI) is obtained with the addition of one more unit operation, such as distillation. Depending on the industrial plants, the obtention of water can vary the production sequence. Proper water treatment is significant for the effectiveness and safety of the process.

### Steam generation

Steam generators of industrial facilities evaluated are classified into three categories: industrial Boiler, Electric Steam Generator (ESG), and Pure Steam Generator (PSG) accoupled at different distances and distinct points of the evaluated process. Boilers are closed vessels that generate steam at pressures from 7 to 10 bar and supply, in addition to autoclaves, utilities that need heating, such as reactor jackets. The supply water is usually potable or softened, and the heating system for evaporation takes place by burning gas or fuel. Electric steam generators are small packed vessels, with PLC control working with a pressure from 3 to 4 bar, coupled to autoclaves, usually fed by reverse osmosis water, purified water (PW), or water for injection (WFI), in which steam generation occurs by electrical heating of electrical resistances in direct contact with water. These types of equipment are considered low-impact installation due to clean operation, to be quiet, and ease to control. Pure steam generators are heat exchangers that generate saturated steam at pressures from approximately 4 bar and are supplied with purified water (PW) or water for injection (WFI) after passing through a heat exchanger or distiller. This type of equipment provides steam used in different industrial stages or for terminal sterilization. In this case, steam generation occurs by exchanging heat with steam from the boiler, which has no contact with the purified water, providing the energy necessary for the water to change from liquid to vapor. One advantage of these types of steam generators is that the quality of the produced steam is constant in terms of pyrogenic contents, independently of pressure and production flow rate.

The steam generators of manufacturing facilities studied in this work varied in types and spatial configuration. They were installed according to the necessity and possibilities of the factories to increase the steam quality. The water quality can affect the interior of the equipment and the quality of the steam generated in the long term, but in this work, no correlation with process efficiency, type of steam generators, or size of the autoclave was founded.

### Non-condensable gas testing

**Fig 1:**
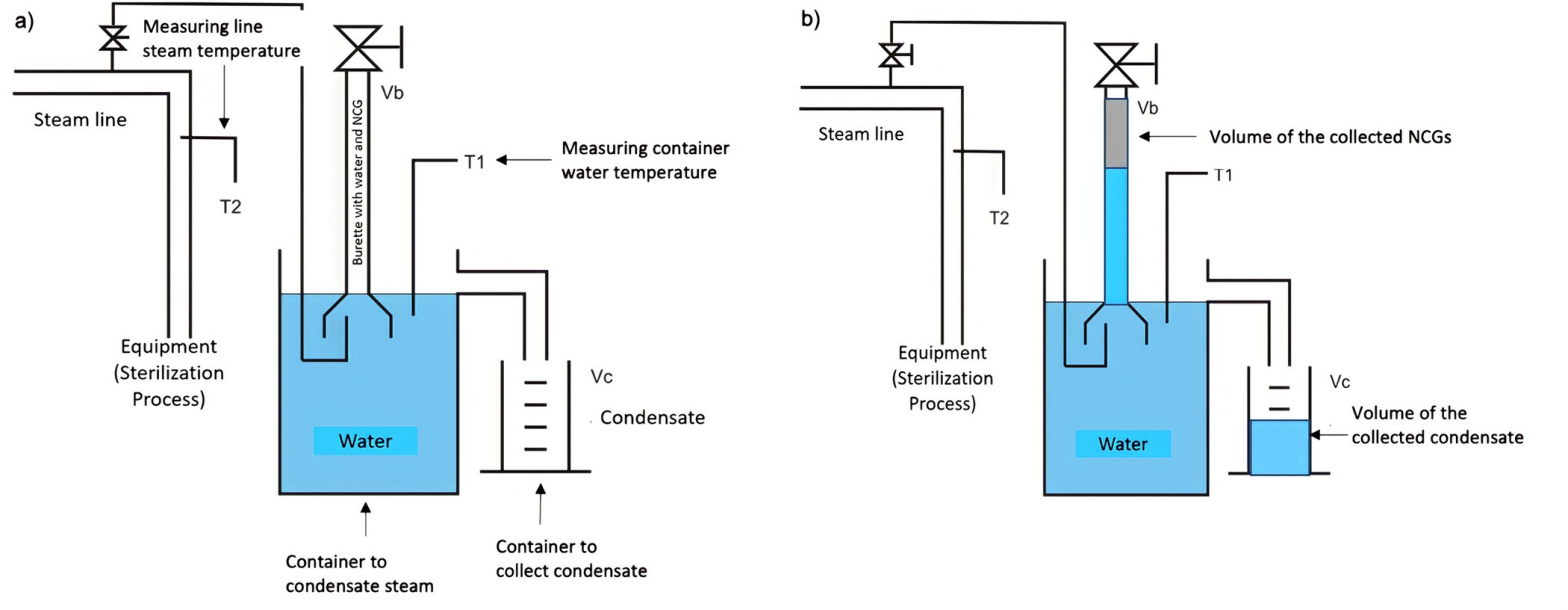
System representation for measuring non-condensable gases (NCG) used in the tests, adapted from EN285:2015, where: a) before the collection, b) after the measurement according to the norm.

An *apparatus* developed and adapted to determine non-condensable gases (Fig 1) as described in EN285:2015 was used to determine the amount of non-condensable gases, NCGs, present in the steam.

A needle valve was connected to the steam pipe evaluated. The system *apparatus* allowed free drainage of condensate through the rubber tubing. The container was filled with cold de-aerated water (water boiled for 5 minutes and cooled to 27°C, previously) until it flowed through the overflow pipe. The burette was filled with cold de-aerated water, inverted, and placed in the container, ensuring that no air was introduced into the burette. All air from the pipe was withdrawn by opening the needle valve, and, with the sampling pipe in the container, cold de-aerated water was added until it flowed through the overflow pipe. A graduated cylinder was positioned under the container overflow, and the steam sampling pipe was located within the funnel. The needle valve was adjusted to allow a continuous sample of steam into the funnel, sufficient to cause a small amount of "steam hammer" to be heard. Ensuring that the steam entering the funnel was discharged, the non-condensable gases were collected in the burette.

The needle valve was closed after first noting the "open" position. A sterilization cycle with the chamber empty started ensuring that the graduated cylinder was empty and the container was filled with water. When the steam supply to the equipment commenced, the needle valve was re-opened, allowing a continuous sample of steam into the funnel sufficient to cause a small amount of "steam hammer" to be heard. The steam sample condensed in the funnel, and the non-condensable gases rose to the top of the burette. The overspill formed by the condensate was collected, and the water was displaced by the gases in the graduated cylinder. The needle valve was closed when the water temperature in the container was between 70°C and 75°C [5].

For NCG determination, the volume (Vb) of water displaced from the burette and the volume (Vc) of water collected in the graduated cylinder, was recorded. For each equipment challenged, determining the percentage of non-condensable gases, according to Eq 1.


$$Cn\;(\%)=\frac{Vb}{\left(Vp-Vb\right)}X\;100$$ (1)

Where:

Cn = is the content of NCGs, in ml per 100mL of condensate from the steam;

Vb = is the volume of water displaced from the burette (mL);

Vp = is the volume of water collected in the graduated cylinder (mL).

All essays for NCGs measurement were performed in triplicate, and the results were considered independent values, not being treated with acceptable ranges.

### Data analysis

The data obtained were analyzed using Minitab^®^ software, version 18.1, to identify a relationship between the water quality used for steam generation and the concentration of non-condensable gases determined into equipment qualification.

## Results

The types of water used in the saturated steam generation systems of the industries evaluated in this study were Softened Water (SW), one-step reverse osmosis (RO), purified water (PW), and water for injection (WFI). Purified water (PW) and water for injection (WFI) were characterized according to the specifications of the pharmaceutical compendiums (Table 1). To obtain these different types of water, it is possible to carry out just one step, such as reverse osmosis, or a sequence of treatments such as, for example, distillation, ion exchange, and reverse osmosis as described in the European Pharmacopoeia. Although these types of water have criteria for Total Organic Carbon (TOC), there is no assess to how much this reflects on the concentration of CNGs present in the steam since gases such as O_2_ and N_2_ are not detected in the TOC analysis.

**Table 1:** Treatment steps and parameters of purified water and purified water for injection or water for injection considering pharmaceutical compendiums [23–26].

	Parameters	Brazilian Pharmacopeia	United States Pharmacopeia	European Pharmacopeia	Japanese Pharmacopeia
**Purified water**	Treatment	Reverse osmosis or a combination of purification techniques from drinking water	Reverse osmosis or a combination of purification techniques from drinking water	Distillation, ion exchange, reverse osmosis, or another suitable process	Distillation, ion exchange, reverse osmosis, ultrafiltration, or another suitable process
	Conductivity (25°C)	max. 1.3 μS/cm	max. 1.3 μS/cm	max. 5.1 μS/cm	max. 1.3 μS/cm
	Heterotrophic bacteria	max. 100 UFC/mL	max. 100 UFC/mL	max. 100 UFC/mL	max. 100 UFC/mL
	Bacterial endotoxin	not included	Not included	<0,25 UE/mL	Not included
	Total Organic Carbon	≤ 0.50 mg/L	≤ 0.50 mg/L	≤ 0.50 mg/L	≤ 0.50 mg/L
**Water for Injection**	Treatment	Distillation or similar process from drinking or purified water	Reverse osmosis or distillation	Distillation	Distillation, reverse osmosis, ultrafiltration, or a techniques combination
	Conductivity (25°C)	max. 1.3 μS/cm	max. 1.3 μS/cm	max. 1.3 μS/cm	max. 1.3 μS/cm
	Heterotrophic bacteria	max. 10 UFC/mL	max. 10 UFC/mL	max. 10 UFC/mL	max. 10 UFC/mL
	Bacterial endotoxin	< 0.25 EU/mL	< 0.25 EU/mL	< 0.25 EU/mL	< 0.25 EU/mL
	Total Organic Carbon	≤ 0.50 mg/L	≤ 0.50 mg/L	≤ 0.50 mg/L	≤ 0.50 mg/L

Although these types of water have criteria for Total Organic Carbon (TOC), we did not assess how much this reflects on the concentration of CNGs present in the steam since gases such as O_2_ and N_2_ are not detected in the TOC analysis.

Water quality parameters controls are evaluated internally and are unique for each company. Acquisition data is just related to the validation processes plan. Parameters such as TOC and conductivity are defined and must be in accordance with Brazilian Authorities Regulation.

Among 170 tests performed, nine were related to equipment that used softened water to generate saturated steam; 14 devices were connected to one-step reverse osmosis water; 68 tests used purified water (PW), and 79 tests used water for injection (WFI) to generate steam. From total tests performed, 109 tests passed, and 61 tests failed, representing 64.2% and 35.8%, respectively. According to EN285:2015, the maximum value collected during the tests must be less than or equal to 3.5%, which means, for every 100mL of condensate collected, a maximum of 3.5mL of NCGs can be presented at steam condensate. Therefore, tests that showed values above 3.5% were considered to have failed. A higher failure rate was observed in terms of concentration of non-condensable gases in systems that used purified water for steam generation (64.7%), followed by softened water (55.6%), one-step reverse osmosis (42.9%), and water for injection (7.6%) (Table 2).

**Table 2:** Non-condensable gases concentration tests performed, approved, or failed tests (%) based on the type of water used for steam generation, from September/16 to August/21.

Water quality	Total Tests		Approved tests		Failed tests	
	(n)	(%)	(n)	(%)	(n)	(%)
Softened	09	5.3	04	44.4	05	55.6
One-step reverse osmosis	14	8.2	08	57.1	06	42.9
Purified Water (PW)	68	40.0	24	35.3	44	64.7
Water for Injection (WFI)	79	46.5	73	92.4	06	7.6
Total	170	100.0	109	64.2	61	35.8

## Discussion

Steam sterilization processes are based on high heat transfer rates from the steam to the load that occurs due to condensation. The presence of NCGs inside the chamber reduces the amount of energy available for latent heat conduction in saturated steam sterilization processes, harming the inactivation of microorganisms and, consequently, decreasing the lethality of the process. Also, the presence of NCGs prevents the contact of the steam and the material, making difficult its condensation on the surface to be sterilized, to exchange the energy necessary to sterilization. Non-condensable gases can be originated from failures in the vacuum performed in the chamber but can also be generated during the process by possible leaks and micro-cracks in the junctions and pipes in the steam piping or inadequate generator degassing [11, 24, 27].

EN285 note that the method indicated in the standard norm is based on possible conditions when the methodology was established (1960). A single measurement is insufficient to predict the real concentration of NCGs present in the steam, requiring more than one collection to estimate the actual amount of NCGs. Currently, other methodologies are being proposed to obtain more precise values, such as Computational Fluids Dynamics (CFD) studies [12–14] or even applying mathematical models based on temperature and pressure measurements inside the sterilizers. In this study, the data collection is connected to the steam supply pipe for the sterilizers, making it difficult to insert temperature and pressure sensors to apply such methods. The EN285 methodology for data collection brings the relationship between the gas concentration in volume found in the steam condensate, with a limit of 3.5mL of NCGs for every 100mL of steam condensate collected at atmospheric pressure, so the maximum limit is 3.5%.

Most of the equipment evaluated (86.5%) used purified water (PW) or water for injection (WFI), which differ from each other by the permitted limit of endotoxins (Table 1), and from the point of view of the method of treatment, by the additional step of water purification for injectables.

In order to test for the equality and the differences between pairs of means, one-way ANOVA (Minitab^®^ version 18.1) was performed to compare four types of water quality used for steam generation.

**Fig 2:**
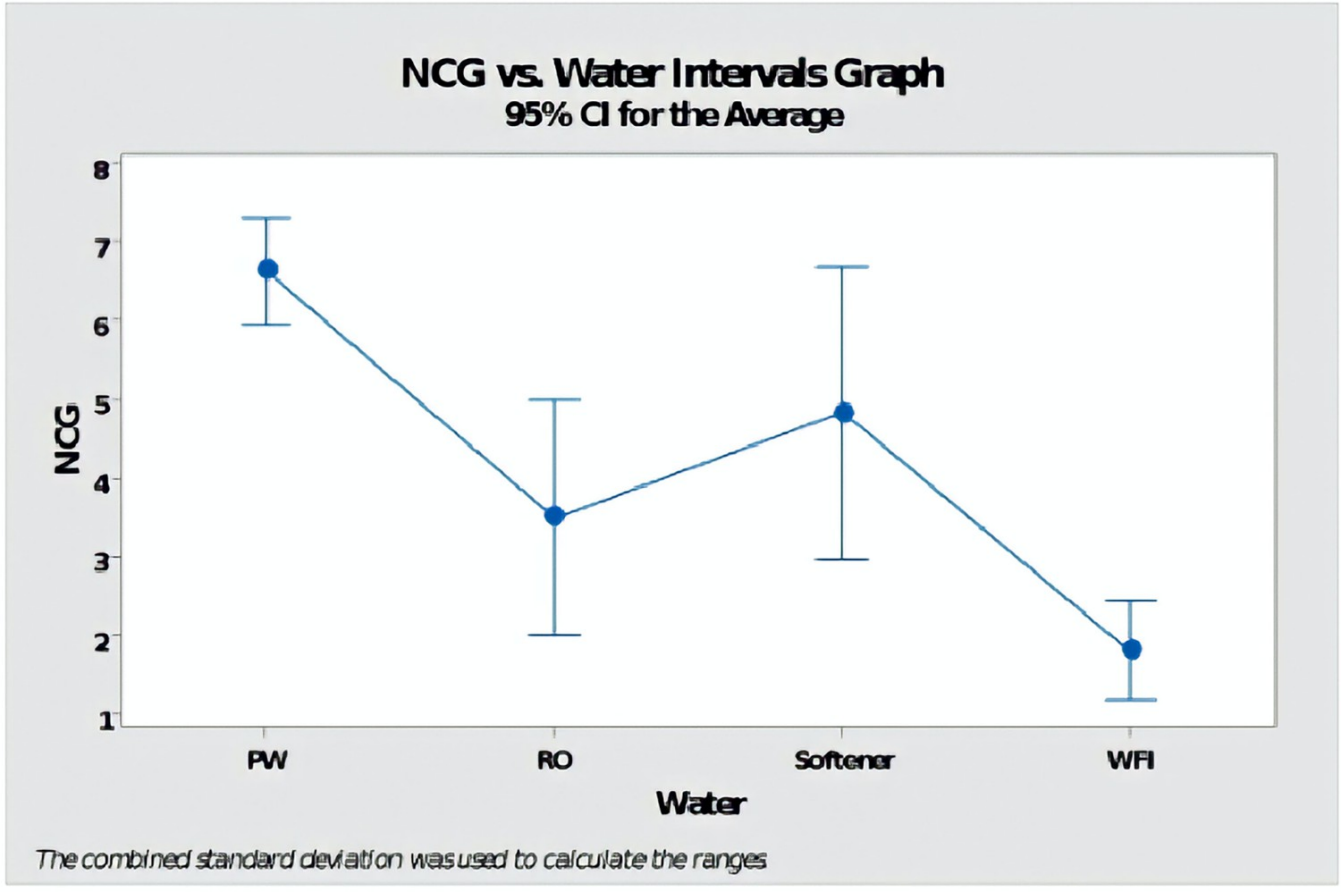
Interval plot of NCGs concentration versus types of water used for steam generation.

Considering the 95% confidence interval of mean water type groups, a one-way ANOVA revealed a statistically significant difference in at least one group (p-value = 0.00) in terms of NCGs concentration. Also, there was an overlap in the confidence intervals between the one-step reverse osmosis and water for injection samples (Fig 2, Table 3).

**Table 3:** Descriptive statistics (mean, standard deviation, and 95% confidence intervals) for each separate water type group analyzed (softened, reverse osmosis, purified, and WFI).

Type of water	Tests (n)	Mean value	Standard Deviation	CI _95%_
SW	27	4.822	4.55	(2.644; 6.149)
RO	42	3.495	2.624	(2.013; 4.978)
PW	204	6.621	7.298	(5.949; 7.294)
WFI	237	1.8105	1.4106	(1.1864; 2.4347)

SW = softened water; RO = one-step reverse osmosis; PW = purified water; WFI = water for injection.

**Fig 3:**
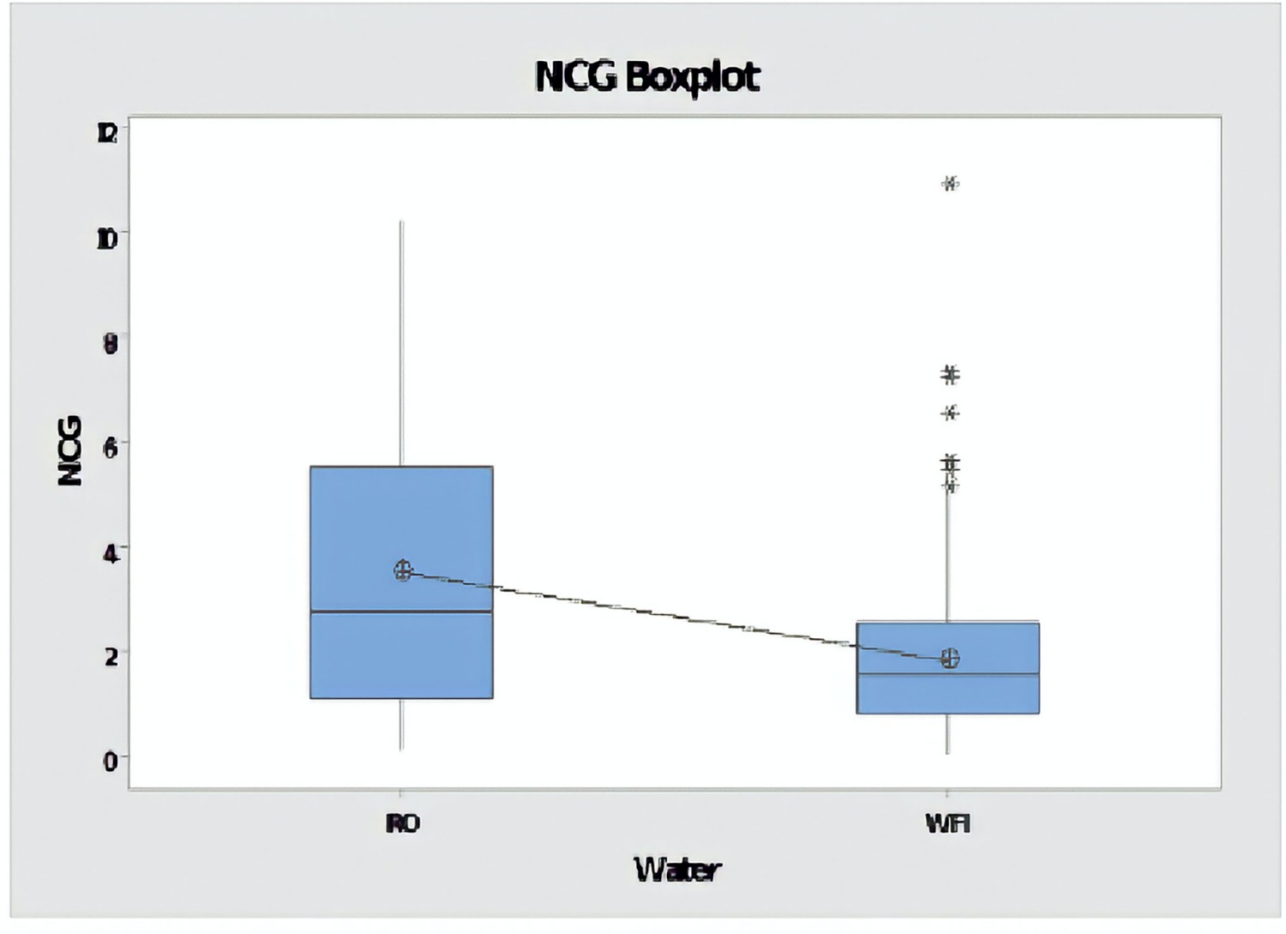
Box-plot of distributions of NCGs concentration comparing tests performed with reverse osmosis water (RO) and water for injection.

A two sample t-test was performed to compare non-condensable gases concentration in RO and WFI. There was a significant difference in NCGs concentration between RO (M = 3.5, SD = 2.62) and WFI (M = 1.81, SD = 1.41); t(45) = 4.06, p = 0.00, proving that type of waters are distinct (Fig 3).

**Fig 4:**
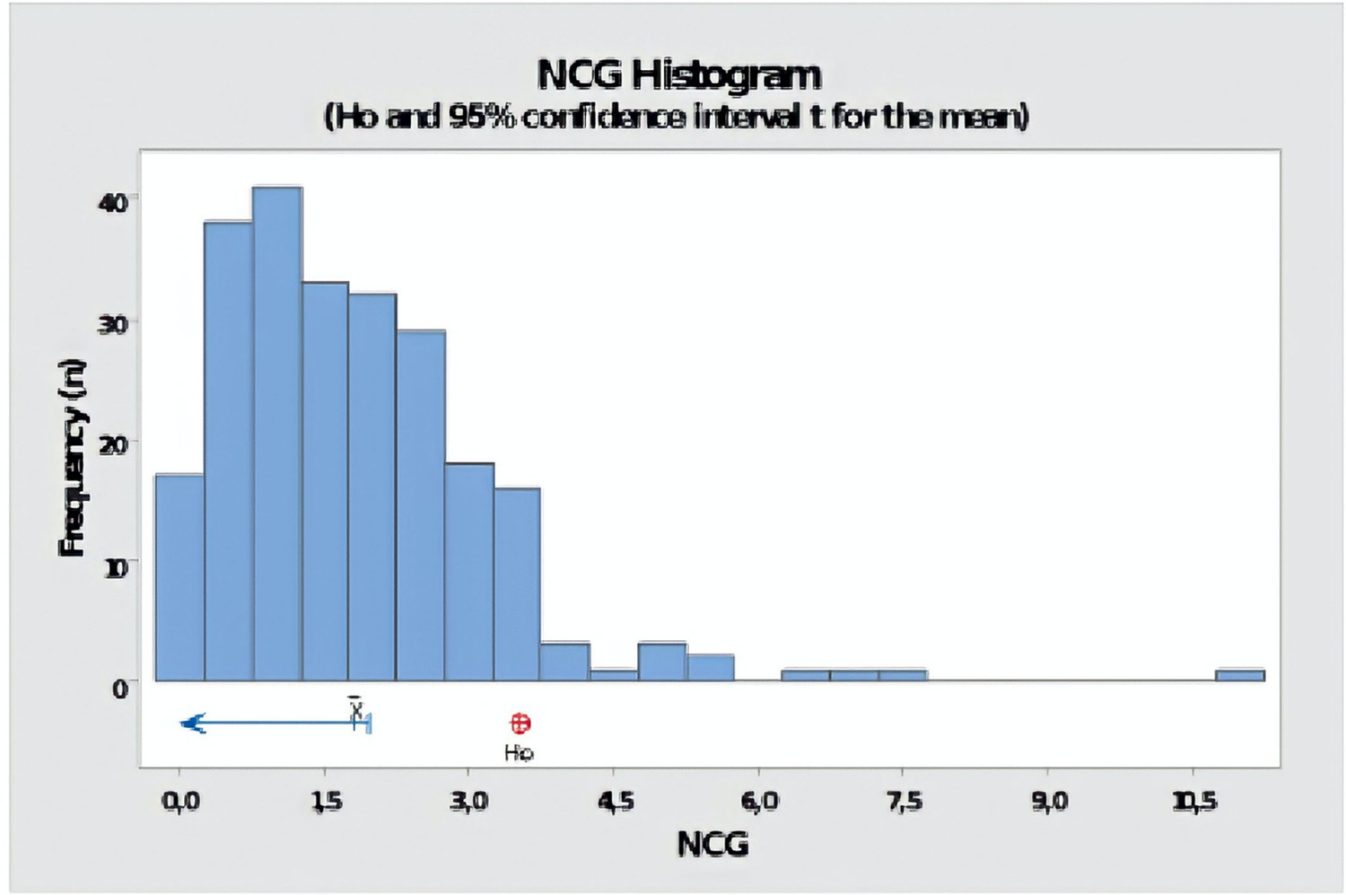
Histogram for NCGs concentration (Ho and 95% CI) for processes using WFI for steam generation.

In the sequence, a one-sample t-test was performed to verify if the set WFI data is below the specified NCGs concentration of 3.5%. It is observed that, with 95% certainty, statistically (p-value = 0.000), the values of GNCs of processes (n = 237 tests) (M = 1.8105, SD = 1.4106) that tests performed using WFI for steam generation are below 3.5% (Ho, Fig 4).

Finally, to evaluate processes systems (equipment + steam generator + water quality), 22 processes systems were defined and submitted to one-way ANOVA, considering all NCGs tests performed (S2 Table). Six of these system processes presented as within 95% CI, NCGs below 3.5% (Table 4). A p-value = 0.000 shows that at least one system differs between them.

**Table 4:** Descriptive statistics (n, mean, standard deviation, and 95% confidence intervals) of process systems with a concentration of NCGs below 3.5%.

Process System	N	Mean	Standard Deviation	95% CI
Autoclave+ ESG +WFI	78	1.760	1.213	(0.736; 2.784)
Autoclave+ PSG +WFI	78	2.240	1.314	(1.216; 3.264)
Bioreactor+ PSG +WFI	12	0.883	1.083	(-1.727; 3.494)
Filling machine+ PSG +WFI	12	0.600	0.457	(-2.010; 3.210)
Reactor+ PSG +WFI	18	1.306	0.847	(-0.826; 3.437)
Tank+PSG+WFI	15	0.893	0.859	(-1.442; 3.228)

PSG = Pure Steam Generator; ESG = Electric Steam Generator; PW = purified water; SW = softened water; RO = reverse osmosis water; WFI = water for injection.

One sample t-test was performed for each process system with NCGs concentration below 3.5%. It is observed values of GNCs of processes: (n = 78 tests) (M = 1.760, SD = 1.2130) to autoclave using WFI for steam generation from ESG.

It is observed values of GNCs of processes: (n = 78 tests) (M = 2.240, SD = 1.3140) to autoclave using WFI; (n = 12 tests) (M = 0.883, SD = 1.083) to bioreactor using WFI; (n = 12 tests) (M = 0.600, SD = 0.457) to filling machine using WFI; (n = 18 tests) (M = 1.306, SD = 0.847) to reactor using WFI and (n = 15 tests) (M = 0.893, SD = 0.859) to tank using WFI. For steam generation these conditions used PSG.

The presence of NCGs has a negative impact on material and patient safety, causing failures in the sterilization cycles, promoting wet loads, damaging materials during terminal sterilization [28]. The water degassing can occur through devices for detection of NCGs or by heating, corroborating the qualification of steam quality [21], although, in Brazil, this requirement is not specified for the equipment [29]. Purified water was kept at 85°C to inhibit microbiological growth, and softened water was maintained at 90°C to reduce the temperature impact for industrial steam generators and boilers supply.

To improve the process and get lower values of NCGs, it is possible to promote the water heating before entering the steam generator, which contributes to the removal of non-condensable gases. The installation of traps along the steam line can avoid condensate from steam, which in contact with saturated steam along the line can form carbonic acid gas (HCO_3_), also characterized as a NCG, which can be incorporated along the line that potentially causes corrosion in the pipeline. Another aspect being considered is that many production lines have leaks, in which, with the high steam velocity in the feed line, outside air can be drawn in by suction, by one phenomenon called the venturi effect. Thus, a preventive maintenance schedule is essential to guarantee an effective sterilization procedure, considering the presence of NCGs.

In pharmaceutical processes control, saturated steam sterilization requires the analysis of condensable gases since thermometric measurements, and microbiological evaluations are not sufficient to guarantee that the safety level of sterility has been reached [30–32].

## Conclusions

System processes using WFI for steam generation showed better results for steam quality approvement, in terms of NCGs concentration, compared to softened, purified, or reverse osmosis water treatments in the concentration of non-condensable gases in steam used for sterilization processes of industrial utilities. Ideally, only treated water (i.e., softened, purified, heated, and degassed) as WFI is recommended for steam generation for sterilization, but it is not an affordable solution to all industrial facilities.

## Supporting information

S1 Table Equipment, steam generator, type of water, NCGs concentration for tests performed. (DOCX)

S2 Table Descriptive statistics (n, mean, standard deviation, and 95% confidence intervals) of process systems were evaluated. (DOCX)

S1 Fig NCGs Histogram for process systems (H_0_ and 95% confidence interval for the mean). (DOCX)
