# Supplementary material for: Analysis of water quality over non-condensable gases concentration on steam used for sterilization
Source: PLoS One. 2022 Sep 27;17(9):e0274924. doi: 10.1371/journal.pone.0274924 (PMC9514632; doi:10.1371/journal.pone.0274924)
Supplement: S1 Table — (DOCX) [file pone.0274924.s001.docx]

S1 Table. Equipment, steam generator, type of water, NCGs concentration for tests performed.

| **Test** | **Equipment** | **Steam** | **Water** | **NCG** |
| --- | --- | --- | --- | --- |
| 1 | Autoclave | Pure Steam Generator | WFI | 3.3 |
| 2 | Autoclave | Pure Steam Generator | WFI | 1.4 |
| 3 | Autoclave | Pure Steam Generator | WFI | 1.1 |
| 4 | Autoclave | Electric Steam Generator | WFI | 3.0 |
| 5 | Autoclave | Electric Steam Generator | WFI | 3.1 |
| 6 | Autoclave | Electric Steam Generator | WFI | 2.5 |
| 7 | Autoclave | Electric Steam Generator | WFI | 1.0 |
| 8 | Autoclave | Pure Steam Generator | WFI | 3.4 |
| 9 | Autoclave | Pure Steam Generator | WFI | 3.5 |
| 10 | Autoclave | Pure Steam Generator | WFI | 0.8 |
| 11 | Autoclave | Pure Steam Generator | WFI | 2.2 |
| 12 | Autoclave | Electric Steam Generator | WFI | 1.7 |
| 13 | Autoclave | Electric Steam Generator | WFI | 0.4 |
| 14 | Autoclave | Electric Steam Generator | WFI | 5.6 |
| 15 | Autoclave | Electric Steam Generator | WFI | 2.3 |
| 16 | Autoclave | Electric Steam Generator | WFI | 3.5 |
| 17 | Autoclave | Pure Steam Generator | WFI | 2.4 |
| 18 | Autoclave | Pure Steam Generator | WFI | 2.2 |
| 19 | Autoclave | Pure Steam Generator | WFI | 1.1 |
| 20 | Autoclave | Electric Steam Generator | WFI | 3.2 |
| 21 | Autoclave | Pure Steam Generator | WFI | 2.8 |
| 22 | Autoclave | Electric Steam Generator | WFI | 4.7 |
| 23 | Autoclave | Electric Steam Generator | WFI | 2.9 |
| 24 | Autoclave | Pure Steam Generator | WFI | 5.0 |
| 25 | Autoclave | Electric Steam Generator | WFI | 2.3 |
| 26 | Autoclave | Electric Steam Generator | WFI | 1.5 |
| 27 | Autoclave | Electric Steam Generator | WFI | 2.4 |
| 28 | Autoclave | Pure Steam Generator | WFI | 3.4 |
| 29 | Autoclave | Pure Steam Generator | WFI | 1.1 |
| 30 | Autoclave | Pure Steam Generator | WFI | 1.4 |
| 31 | Autoclave | Pure Steam Generator | WFI | 6.5 |
| 32 | Autoclave | Electric Steam Generator | WFI | 1.3 |
| 33 | Autoclave | Pure Steam Generator | WFI | 2.5 |
| 34 | Autoclave | Pure Steam Generator | WFI | 0.8 |
| 35 | Autoclave | Pure Steam Generator | WFI | 1.1 |
| 36 | Autoclave | Pure Steam Generator | WFI | 1.0 |
| 37 | Autoclave | Pure Steam Generator | WFI | 1.7 |
| 38 | Autoclave | Electric Steam Generator | WFI | 1.0 |
| 39 | Autoclave | Electric Steam Generator | WFI | 1.8 |
| 40 | Autoclave | Electric Steam Generator | WFI | 1.1 |
| 41 | Autoclave | Pure Steam Generator | WFI | 3.8 |
| 42 | Autoclave | Pure Steam Generator | WFI | 3.5 |
| 43 | Autoclave | Pure Steam Generator | WFI | 2.7 |
| 44 | Autoclave | Electric Steam Generator | WFI | 1.9 |
| 45 | Autoclave | Electric Steam Generator | WFI | 0.2 |
| 46 | Autoclave | Electric Steam Generator | WFI | 3.1 |
| 47 | Autoclave | Electric Steam Generator | WFI | 0.3 |
| 48 | Autoclave | Electric Steam Generator | WFI | 0.2 |
| 49 | Autoclave | Electric Steam Generator | WFI | 1.5 |
| 50 | Autoclave | Electric Steam Generator | WFI | 0.7 |
| 51 | Autoclave | Pure Steam Generator | WFI | 3.1 |
| 52 | Autoclave | Pure Steam Generator | WFI | 2.3 |
| 53 | Autoclave | Pure Steam Generator | WFI | 2.0 |
| 54 | Autoclave | Pure Steam Generator | WFI | 0.6 |
| 55 | Autoclave | Pure Steam Generator | WFI | 2.4 |
| 56 | Autoclave | Electric Steam Generator | WFI | 2.2 |
| 57 | Autoclave | Electric Steam Generator | WFI | 3.5 |
| 58 | Autoclave | Electric Steam Generator | WFI | 2.7 |
| 59 | Autoclave | Electric Steam Generator | WFI | 3.4 |
| 60 | Autoclave | Pure Steam Generator | WFI | 3.4 |
| 61 | Autoclave | Pure Steam Generator | WFI | 2.5 |
| 62 | Autoclave | Pure Steam Generator | WFI | 0.8 |
| 63 | Autoclave | Pure Steam Generator | WFI | 1.3 |
| 64 | Autoclave | Electric Steam Generator | WFI | 2.5 |
| 65 | Autoclave | Electric Steam Generator | WFI | 3.0 |
| 66 | Autoclave | Electric Steam Generator | WFI | 3.4 |
| 67 | Autoclave | Electric Steam Generator | WFI | 0.4 |
| 68 | Autoclave | Electric Steam Generator | WFI | 1.9 |
| 69 | Autoclave | Pure Steam Generator | WFI | 1.9 |
| 70 | Autoclave | Pure Steam Generator | WFI | 0.9 |
| 71 | Autoclave | Pure Steam Generator | WFI | 1.9 |
| 72 | Autoclave | Electric Steam Generator | WFI | 1.4 |
| 73 | Autoclave | Pure Steam Generator | WFI | 3.5 |
| 74 | Autoclave | Electric Steam Generator | WFI | 3.9 |
| 75 | Autoclave | Electric Steam Generator | WFI | 0.9 |
| 76 | Autoclave | Pure Steam Generator | WFI | 2.0 |
| 77 | Autoclave | Electric Steam Generator | WFI | 0.5 |
| 78 | Autoclave | Electric Steam Generator | WFI | 0.9 |
| 79 | Autoclave | Electric Steam Generator | WFI | 1.1 |
| 80 | Autoclave | Pure Steam Generator | WFI | 2.0 |
| 81 | Autoclave | Pure Steam Generator | WFI | 2.0 |
| 82 | Autoclave | Pure Steam Generator | WFI | 2.5 |
| 83 | Autoclave | Pure Steam Generator | WFI | 3.8 |
| 84 | Autoclave | Electric Steam Generator | WFI | 2.0 |
| 85 | Autoclave | Pure Steam Generator | WFI | 2.8 |
| 86 | Autoclave | Pure Steam Generator | WFI | 0.5 |
| 87 | Autoclave | Pure Steam Generator | WFI | 2.1 |
| 88 | Autoclave | Pure Steam Generator | WFI | 1.9 |
| 89 | Autoclave | Pure Steam Generator | WFI | 1.0 |
| 90 | Autoclave | Electric Steam Generator | WFI | 1.0 |
| 91 | Autoclave | Electric Steam Generator | WFI | 2.5 |
| 92 | Autoclave | Electric Steam Generator | WFI | 1.0 |
| 93 | Autoclave | Pure Steam Generator | WFI | 0.5 |
| 94 | Autoclave | Pure Steam Generator | WFI | 3.0 |
| 95 | Autoclave | Pure Steam Generator | WFI | 1.6 |
| 96 | Autoclave | Electric Steam Generator | WFI | 0.5 |
| 97 | Autoclave | Electric Steam Generator | WFI | 0.4 |
| 98 | Autoclave | Electric Steam Generator | WFI | 1.1 |
| 99 | Autoclave | Electric Steam Generator | WFI | 0.7 |
| 100 | Autoclave | Electric Steam Generator | WFI | 0.7 |
| 101 | Autoclave | Electric Steam Generator | WFI | 1.5 |
| 102 | Autoclave | Electric Steam Generator | WFI | 0.6 |
| 103 | Autoclave | Pure Steam Generator | WFI | 1.2 |
| 104 | Autoclave | Pure Steam Generator | WFI | 1.9 |
| 105 | Autoclave | Pure Steam Generator | WFI | 1.4 |
| 106 | Autoclave | Pure Steam Generator | WFI | 2.4 |
| 107 | Autoclave | Pure Steam Generator | WFI | 1.3 |
| 108 | Autoclave | Electric Steam Generator | WFI | 2.2 |
| 109 | Autoclave | Electric Steam Generator | WFI | 2.8 |
| 110 | Autoclave | Electric Steam Generator | WFI | 1.3 |
| 111 | Autoclave | Electric Steam Generator | WFI | 0.9 |
| 112 | Autoclave | Pure Steam Generator | WFI | 3.3 |
| 113 | Autoclave | Pure Steam Generator | WFI | 2.0 |
| 114 | Autoclave | Pure Steam Generator | WFI | 2.4 |
| 115 | Autoclave | Pure Steam Generator | WFI | 1.5 |
| 116 | Autoclave | Electric Steam Generator | WFI | 2.2 |
| 117 | Autoclave | Electric Steam Generator | WFI | 1.9 |
| 118 | Autoclave | Electric Steam Generator | WFI | 2.7 |
| 119 | Autoclave | Electric Steam Generator | WFI | 0.3 |
| 120 | Autoclave | Electric Steam Generator | WFI | 3.3 |
| 121 | Autoclave | Pure Steam Generator | WFI | 1.7 |
| 122 | Autoclave | Pure Steam Generator | WFI | 0.7 |
| 123 | Autoclave | Pure Steam Generator | WFI | 1.4 |
| 124 | Autoclave | Electric Steam Generator | WFI | 1.5 |
| 125 | Autoclave | Pure Steam Generator | WFI | 1.9 |
| 126 | Autoclave | Electric Steam Generator | WFI | 4.9 |
| 127 | Autoclave | Electric Steam Generator | WFI | 2.2 |
| 128 | Autoclave | Pure Steam Generator | WFI | 5.1 |
| 129 | Autoclave | Electric Steam Generator | WFI | 1.2 |
| 130 | Autoclave | Electric Steam Generator | WFI | 0.9 |
| 131 | Autoclave | Electric Steam Generator | WFI | 2.3 |
| 132 | Autoclave | Pure Steam Generator | WFI | 3.1 |
| 133 | Autoclave | Pure Steam Generator | WFI | 3.0 |
| 134 | Autoclave | Pure Steam Generator | WFI | 1.6 |
| 135 | Autoclave | Pure Steam Generator | WFI | 5.4 |
| 136 | Autoclave | Electric Steam Generator | WFI | 3.1 |
| 137 | Autoclave | Pure Steam Generator | WFI | 2.8 |
| 138 | Autoclave | Pure Steam Generator | WFI | 0.5 |
| 139 | Autoclave | Pure Steam Generator | WFI | 1.7 |
| 140 | Autoclave | Pure Steam Generator | WFI | 0.3 |
| 141 | Autoclave | Pure Steam Generator | WFI | 1.5 |
| 142 | Autoclave | Electric Steam Generator | WFI | 0.7 |
| 143 | Autoclave | Electric Steam Generator | WFI | 0.5 |
| 144 | Autoclave | Electric Steam Generator | WFI | 0.9 |
| 145 | Autoclave | Pure Steam Generator | WFI | 7.2 |
| 146 | Autoclave | Pure Steam Generator | WFI | 1.4 |
| 147 | Autoclave | Pure Steam Generator | WFI | 2.0 |
| 148 | Autoclave | Electric Steam Generator | WFI | 1.2 |
| 149 | Autoclave | Electric Steam Generator | WFI | 0.3 |
| 150 | Autoclave | Electric Steam Generator | WFI | 0.8 |
| 151 | Autoclave | Electric Steam Generator | WFI | 0.5 |
| 152 | Autoclave | Electric Steam Generator | WFI | 0.5 |
| 153 | Autoclave | Electric Steam Generator | WFI | 1.1 |
| 154 | Autoclave | Electric Steam Generator | WFI | 0.2 |
| 155 | Autoclave | Pure Steam Generator | WFI | 2.5 |
| 156 | Autoclave | Pure Steam Generator | WFI | 2.5 |
| 157 | Bioreactor | Pure Steam Generator | WFI | 2.9 |
| 158 | Bioreactor | Pure Steam Generator | WFI | 0.5 |
| 159 | Bioreactor | Pure Steam Generator | WFI | 0.9 |
| 160 | Bioreactor | Pure Steam Generator | WFI | 0.1 |
| 161 | Bioreactor | Pure Steam Generator | WFI | 1.3 |
| 162 | Bioreactor | Pure Steam Generator | WFI | 0.2 |
| 163 | Bioreactor | Pure Steam Generator | WFI | 0.0 |
| 164 | Bioreactor | Pure Steam Generator | WFI | 0.3 |
| 165 | Bioreactor | Pure Steam Generator | WFI | 0.6 |
| 166 | Bioreactor | Pure Steam Generator | WFI | 0.0 |
| 167 | Bioreactor | Pure Steam Generator | WFI | 0.6 |
| 168 | Bioreactor | Pure Steam Generator | WFI | 3.2 |
| 169 | Filling machine | Pure Steam Generator | WFI | 0.0 |
| 170 | Filling machine | Pure Steam Generator | WFI | 0.6 |
| 171 | Filling machine | Pure Steam Generator | WFI | 0.6 |
| 172 | Filling machine | Pure Steam Generator | WFI | 1.2 |
| 173 | Filling machine | Pure Steam Generator | WFI | 0.2 |
| 174 | Filling machine | Pure Steam Generator | WFI | 0.3 |
| 175 | Filling machine | Pure Steam Generator | WFI | 0.9 |
| 176 | Filling machine | Pure Steam Generator | WFI | 1.3 |
| 177 | Filling machine | Pure Steam Generator | WFI | 0.1 |
| 178 | Filling machine | Pure Steam Generator | WFI | 0.1 |
| 179 | Filling machine | Pure Steam Generator | WFI | 1.0 |
| 180 | Filling machine | Pure Steam Generator | WFI | 0.9 |
| 181 | Steam Generator | Pure Steam Generator | WFI | 10.9 |
| 182 | Steam Generator | Pure Steam Generator | WFI | 1.8 |
| 183 | Steam Generator | Pure Steam Generator | WFI | 0.9 |
| 184 | Steam Generator | Pure Steam Generator | WFI | 3.3 |
| 185 | Steam Generator | Pure Steam Generator | WFI | 2.2 |
| 186 | Steam Generator | Pure Steam Generator | WFI | 2.4 |
| 187 | Steam Generator | Pure Steam Generator | WFI | 7.3 |
| 188 | Steam Generator | Pure Steam Generator | WFI | 2.1 |
| 189 | Steam Generator | Pure Steam Generator | WFI | 0.7 |
| 190 | Homogenizer | Pure Steam Generator | WFI | 2.6 |
| 191 | Homogenizer | Pure Steam Generator | WFI | 2.0 |
| 192 | Homogenizer | Pure Steam Generator | WFI | 2.6 |
| 193 | Freeze dryer | Pure Steam Generator | WFI | 1.5 |
| 194 | Freeze dryer | Pure Steam Generator | WFI | 1.0 |
| 195 | Freeze dryer | Pure Steam Generator | WFI | 2.9 |
| 196 | Sterilization point | Pure Steam Generator | WFI | 2.7 |
| 197 | Sterilization point | Pure Steam Generator | WFI | 1.8 |
| 198 | Sterilization point | Pure Steam Generator | WFI | 3.4 |
| 199 | Sterilization point | Pure Steam Generator | WFI | 2.3 |
| 200 | Sterilization point | Pure Steam Generator | WFI | 1.7 |
| 201 | Sterilization point | Pure Steam Generator | WFI | 2.5 |
| 202 | Sterilization point | Pure Steam Generator | WFI | 1.3 |
| 203 | Sterilization point | Pure Steam Generator | WFI | 1.5 |
| 204 | Sterilization point | Pure Steam Generator | WFI | 1.0 |
| 205 | Reactor | Pure Steam Generator | WFI | 0.8 |
| 206 | Reactor | Pure Steam Generator | WFI | 1.0 |
| 207 | Reactor | Pure Steam Generator | WFI | 1.9 |
| 208 | Reactor | Pure Steam Generator | WFI | 1.8 |
| 209 | Reactor | Pure Steam Generator | WFI | 2.5 |
| 210 | Reactor | Pure Steam Generator | WFI | 0.2 |
| 211 | Reactor | Pure Steam Generator | WFI | 0.2 |
| 212 | Reactor | Pure Steam Generator | WFI | 0.5 |
| 213 | Reactor | Pure Steam Generator | WFI | 1.4 |
| 214 | Reactor | Pure Steam Generator | WFI | 2.2 |
| 215 | Reactor | Pure Steam Generator | WFI | 1.0 |
| 216 | Reactor | Pure Steam Generator | WFI | 3.3 |
| 217 | Reactor | Pure Steam Generator | WFI | 1.6 |
| 218 | Reactor | Pure Steam Generator | WFI | 1.6 |
| 219 | Reactor | Pure Steam Generator | WFI | 1.5 |
| 220 | Reactor | Pure Steam Generator | WFI | 0.7 |
| 221 | Reactor | Pure Steam Generator | WFI | 1.1 |
| 222 | Reactor | Pure Steam Generator | WFI | 0.2 |
| 223 | Tank | Pure Steam Generator | WFI | 0.3 |
| 224 | Tank | Pure Steam Generator | WFI | 0.7 |
| 225 | Tank | Pure Steam Generator | WFI | 0.5 |
| 226 | Tank | Pure Steam Generator | WFI | 2.3 |
| 227 | Tank | Pure Steam Generator | WFI | 0.3 |
| 228 | Tank | Pure Steam Generator | WFI | 0.3 |
| 229 | Tank | Pure Steam Generator | WFI | 0.0 |
| 230 | Tank | Pure Steam Generator | WFI | 0.1 |
| 231 | Tank | Pure Steam Generator | WFI | 1.2 |
| 232 | Tank | Pure Steam Generator | WFI | 2.6 |
| 233 | Tank | Pure Steam Generator | WFI | 1.5 |
| 234 | Tank | Pure Steam Generator | WFI | 0.2 |
| 235 | Tank | Pure Steam Generator | WFI | 0.3 |
| 236 | Tank | Pure Steam Generator | WFI | 2.1 |
| 237 | Tank | Pure Steam Generator | WFI | 1.0 |
| 238 | Autoclave | Pure Steam Generator | PW | 6.0 |
| 239 | Autoclave | Pure Steam Generator | PW | 2.1 |
| 240 | Autoclave | Pure Steam Generator | PW | 0.3 |
| 241 | Autoclave | Pure Steam Generator | PW | 5.8 |
| 242 | Autoclave | Pure Steam Generator | PW | 7.1 |
| 243 | Autoclave | Electric Steam Generator | PW | 1.7 |
| 244 | Autoclave | Electric Steam Generator | PW | 2.6 |
| 245 | Autoclave | Pure Steam Generator | PW | 8.1 |
| 246 | Autoclave | Pure Steam Generator | PW | 16.5 |
| 247 | Autoclave | Pure Steam Generator | PW | 14.0 |
| 248 | Autoclave | Pure Steam Generator | PW | 7.7 |
| 249 | Autoclave | Pure Steam Generator | PW | 6.4 |
| 250 | Autoclave | Electric Steam Generator | PW | 4.3 |
| 251 | Autoclave | Pure Steam Generator | PW | 3.0 |
| 252 | Autoclave | Pure Steam Generator | PW | 3.1 |
| 253 | Autoclave | Electric Steam Generator | PW | 16.7 |
| 254 | Autoclave | Pure Steam Generator | PW | 5.1 |
| 255 | Autoclave | Pure Steam Generator | PW | 10.4 |
| 256 | Autoclave | Electric Steam Generator | PW | 8.1 |
| 257 | Autoclave | Electric Steam Generator | PW | 4.3 |
| 258 | Autoclave | Electric Steam Generator | PW | 1.9 |
| 259 | Autoclave | Electric Steam Generator | PW | 3.4 |
| 260 | Autoclave | Electric Steam Generator | PW | 1.4 |
| 261 | Autoclave | Electric Steam Generator | PW | 3.2 |
| 262 | Autoclave | Electric Steam Generator | PW | 0.5 |
| 263 | Autoclave | Pure Steam Generator | PW | 0.5 |
| 264 | Autoclave | Pure Steam Generator | PW | 5.9 |
| 265 | Autoclave | Pure Steam Generator | PW | 7.8 |
| 266 | Autoclave | Pure Steam Generator | PW | 6.7 |
| 267 | Autoclave | Electric Steam Generator | PW | 1.4 |
| 268 | Autoclave | Pure Steam Generator | PW | 0.9 |
| 269 | Autoclave | Pure Steam Generator | PW | 4.1 |
| 270 | Autoclave | Electric Steam Generator | PW | 2.7 |
| 271 | Autoclave | Pure Steam Generator | PW | 4.8 |
| 272 | Autoclave | Electric Steam Generator | PW | 3.2 |
| 273 | Autoclave | Electric Steam Generator | PW | 1.1 |
| 274 | Autoclave | Pure Steam Generator | PW | 13.8 |
| 275 | Autoclave | Pure Steam Generator | PW | 4.0 |
| 276 | Autoclave | Pure Steam Generator | PW | 31.9 |
| 277 | Autoclave | Pure Steam Generator | PW | 19.2 |
| 278 | Autoclave | Electric Steam Generator | PW | 15.5 |
| 279 | Autoclave | Electric Steam Generator | PW | 4.7 |
| 280 | Autoclave | Electric Steam Generator | PW | 1.6 |
| 281 | Autoclave | Pure Steam Generator | PW | 0.8 |
| 282 | Autoclave | Pure Steam Generator | PW | 7.7 |
| 283 | Autoclave | Electric Steam Generator | PW | 9.8 |
| 284 | Autoclave | Pure Steam Generator | PW | 4.0 |
| 285 | Autoclave | Pure Steam Generator | PW | 8.4 |
| 286 | Autoclave | Pure Steam Generator | PW | 2.9 |
| 287 | Autoclave | Pure Steam Generator | PW | 0.4 |
| 288 | Autoclave | Pure Steam Generator | PW | 7.0 |
| 289 | Autoclave | Pure Steam Generator | PW | 7.3 |
| 290 | Autoclave | Electric Steam Generator | PW | 0.5 |
| 291 | Autoclave | Electric Steam Generator | PW | 0.3 |
| 292 | Autoclave | Pure Steam Generator | PW | 9.0 |
| 293 | Autoclave | Pure Steam Generator | PW | 7.3 |
| 294 | Autoclave | Pure Steam Generator | PW | 7.8 |
| 295 | Autoclave | Pure Steam Generator | PW | 5.6 |
| 296 | Autoclave | Pure Steam Generator | PW | 3.8 |
| 297 | Autoclave | Electric Steam Generator | PW | 3.3 |
| 298 | Autoclave | Pure Steam Generator | PW | 3.1 |
| 299 | Autoclave | Pure Steam Generator | PW | 1.3 |
| 300 | Autoclave | Electric Steam Generator | PW | 2.4 |
| 301 | Autoclave | Pure Steam Generator | PW | 6.4 |
| 302 | Autoclave | Pure Steam Generator | PW | 1.7 |
| 303 | Autoclave | Electric Steam Generator | PW | 2.4 |
| 304 | Autoclave | Electric Steam Generator | PW | 13.9 |
| 305 | Autoclave | Electric Steam Generator | PW | 1.4 |
| 306 | Autoclave | Electric Steam Generator | PW | 1.0 |
| 307 | Autoclave | Electric Steam Generator | PW | 2.0 |
| 308 | Autoclave | Electric Steam Generator | PW | 0.9 |
| 309 | Autoclave | Electric Steam Generator | PW | 1.7 |
| 310 | Autoclave | Pure Steam Generator | PW | 3.1 |
| 311 | Autoclave | Pure Steam Generator | PW | 4.3 |
| 312 | Autoclave | Pure Steam Generator | PW | 6.7 |
| 313 | Autoclave | Pure Steam Generator | PW | 9.2 |
| 314 | Autoclave | Electric Steam Generator | PW | 0.4 |
| 315 | Autoclave | Pure Steam Generator | PW | 1.3 |
| 316 | Autoclave | Pure Steam Generator | PW | 1.0 |
| 317 | Autoclave | Electric Steam Generator | PW | 3.0 |
| 318 | Autoclave | Pure Steam Generator | PW | 2.7 |
| 319 | Autoclave | Electric Steam Generator | PW | 2.0 |
| 320 | Autoclave | Electric Steam Generator | PW | 0.6 |
| 321 | Autoclave | Pure Steam Generator | PW | 6.5 |
| 322 | Autoclave | Pure Steam Generator | PW | 8.0 |
| 323 | Autoclave | Pure Steam Generator | PW | 38.4 |
| 324 | Autoclave | Pure Steam Generator | PW | 31.4 |
| 325 | Autoclave | Electric Steam Generator | PW | 1.0 |
| 326 | Autoclave | Electric Steam Generator | PW | 5.2 |
| 327 | Autoclave | Electric Steam Generator | PW | 0.8 |
| 328 | Autoclave | Pure Steam Generator | PW | 0.9 |
| 329 | Autoclave | Pure Steam Generator | PW | 9.3 |
| 330 | Autoclave | Electric Steam Generator | PW | 8.8 |
| 331 | Autoclave | Pure Steam Generator | PW | 6.4 |
| 332 | Autoclave | Pure Steam Generator | PW | 5.5 |
| 333 | Autoclave | Pure Steam Generator | PW | 2.8 |
| 334 | Autoclave | Pure Steam Generator | PW | 0.9 |
| 335 | Autoclave | Pure Steam Generator | PW | 7.1 |
| 336 | Autoclave | Pure Steam Generator | PW | 6.2 |
| 337 | Autoclave | Electric Steam Generator | PW | 2.6 |
| 338 | Autoclave | Electric Steam Generator | PW | 2.8 |
| 339 | Autoclave | Pure Steam Generator | PW | 8.1 |
| 340 | Autoclave | Pure Steam Generator | PW | 9.2 |
| 341 | Autoclave | Pure Steam Generator | PW | 11.8 |
| 342 | Autoclave | Pure Steam Generator | PW | 6.5 |
| 343 | Autoclave | Pure Steam Generator | PW | 7.8 |
| 344 | Autoclave | Electric Steam Generator | PW | 11.6 |
| 345 | Autoclave | Pure Steam Generator | PW | 3.5 |
| 346 | Autoclave | Pure Steam Generator | PW | 2.7 |
| 347 | Autoclave | Electric Steam Generator | PW | 4.4 |
| 348 | Autoclave | Pure Steam Generator | PW | 5.1 |
| 349 | Autoclave | Pure Steam Generator | PW | 4.0 |
| 350 | Autoclave | Electric Steam Generator | PW | 8.1 |
| 351 | Autoclave | Electric Steam Generator | PW | 6.8 |
| 352 | Autoclave | Electric Steam Generator | PW | 1.0 |
| 353 | Autoclave | Electric Steam Generator | PW | 1.6 |
| 354 | Autoclave | Electric Steam Generator | PW | 1.4 |
| 355 | Autoclave | Electric Steam Generator | PW | 1.0 |
| 356 | Autoclave | Electric Steam Generator | PW | 1.4 |
| 357 | Autoclave | Pure Steam Generator | PW | 2.5 |
| 358 | Autoclave | Pure Steam Generator | PW | 4.8 |
| 359 | Autoclave | Pure Steam Generator | PW | 6.8 |
| 360 | Autoclave | Pure Steam Generator | PW | 3.8 |
| 361 | Autoclave | Electric Steam Generator | PW | 0.6 |
| 362 | Autoclave | Pure Steam Generator | PW | 2.4 |
| 363 | Autoclave | Pure Steam Generator | PW | 9.3 |
| 364 | Autoclave | Electric Steam Generator | PW | 2.2 |
| 365 | Autoclave | Pure Steam Generator | PW | 8.7 |
| 366 | Autoclave | Electric Steam Generator | PW | 1.2 |
| 367 | Autoclave | Electric Steam Generator | PW | 0.5 |
| 368 | Autoclave | Pure Steam Generator | PW | 11.4 |
| 369 | Autoclave | Pure Steam Generator | PW | 14.2 |
| 370 | Autoclave | Pure Steam Generator | PW | 42.3 |
| 371 | Autoclave | Pure Steam Generator | PW | 37.8 |
| 372 | Autoclave | Electric Steam Generator | PW | 6.9 |
| 373 | Autoclave | Electric Steam Generator | PW | 5.7 |
| 374 | Autoclave | Electric Steam Generator | PW | 2.8 |
| 375 | Autoclave | Pure Steam Generator | PW | 1.9 |
| 376 | Autoclave | Pure Steam Generator | PW | 7.4 |
| 377 | Autoclave | Electric Steam Generator | PW | 2.8 |
| 378 | Autoclave | Pure Steam Generator | PW | 12.9 |
| 379 | Steam Generator | Pure Steam Generator | PW | 9.3 |
| 380 | Steam Generator | Pure Steam Generator | PW | 13.4 |
| 381 | Steam Generator | Pure Steam Generator | PW | 4.2 |
| 382 | Steam Generator | Pure Steam Generator | PW | 31.6 |
| 383 | Steam Generator | Pure Steam Generator | PW | 12.7 |
| 384 | Steam Generator | Pure Steam Generator | PW | 4.3 |
| 385 | Steam Generator | Pure Steam Generator | PW | 4.2 |
| 386 | Steam Generator | Pure Steam Generator | PW | 33.3 |
| 387 | Steam Generator | Pure Steam Generator | PW | 9.1 |
| 388 | Steam Generator | Pure Steam Generator | PW | 6.6 |
| 389 | Steam Generator | Pure Steam Generator | PW | 4.5 |
| 390 | Steam Generator | Pure Steam Generator | PW | 42.9 |
| 391 | Freeze dryer | Pure Steam Generator | PW | 5.3 |
| 392 | Freeze dryer | Pure Steam Generator | PW | 5.2 |
| 393 | Freeze dryer | Pure Steam Generator | PW | 3.2 |
| 394 | Freeze dryer | Pure Steam Generator | PW | 13.0 |
| 395 | Freeze dryer | Pure Steam Generator | PW | 5.2 |
| 396 | Freeze dryer | Pure Steam Generator | PW | 2.2 |
| 397 | Sterilization point | Pure Steam Generator | PW | 10.3 |
| 398 | Sterilization point | Pure Steam Generator | PW | 0.9 |
| 399 | Sterilization point | Pure Steam Generator | PW | 2.7 |
| 400 | Sterilization point | Pure Steam Generator | PW | 7.6 |
| 401 | Sterilization point | Pure Steam Generator | PW | 13.2 |
| 402 | Sterilization point | Pure Steam Generator | PW | 5.6 |
| 403 | Sterilization point | Pure Steam Generator | PW | 1.4 |
| 404 | Sterilization point | Pure Steam Generator | PW | 3.1 |
| 405 | Sterilization point | Pure Steam Generator | PW | 1.3 |
| 406 | Sterilization point | Pure Steam Generator | PW | 12.7 |
| 407 | Sterilization point | Pure Steam Generator | PW | 16.2 |
| 408 | Sterilization point | Pure Steam Generator | PW | 2.8 |
| 409 | Sterilization point | Pure Steam Generator | PW | 9.3 |
| 410 | Sterilization point | Pure Steam Generator | PW | 1.7 |
| 411 | Sterilization point | Pure Steam Generator | PW | 3.4 |
| 412 | Sterilization point | Pure Steam Generator | PW | 5.3 |
| 413 | Sterilization point | Pure Steam Generator | PW | 10.4 |
| 414 | Sterilization point | Pure Steam Generator | PW | 5.2 |
| 415 | Reactor | Pure Steam Generator | PW | 3.0 |
| 416 | Reactor | Pure Steam Generator | PW | 4.6 |
| 417 | Reactor | Pure Steam Generator | PW | 5.3 |
| 418 | Reactor | Pure Steam Generator | PW | 6.7 |
| 419 | Reactor | Pure Steam Generator | PW | 2.6 |
| 420 | Reactor | Pure Steam Generator | PW | 4.9 |
| 421 | Reactor | Pure Steam Generator | PW | 3.2 |
| 422 | Reactor | Pure Steam Generator | PW | 10.7 |
| 423 | Reactor | Pure Steam Generator | PW | 3.3 |
| 424 | Reactor | Pure Steam Generator | PW | 7.0 |
| 425 | Reactor | Pure Steam Generator | PW | 8.7 |
| 426 | Reactor | Pure Steam Generator | PW | 7.0 |
| 427 | Tank | Pure Steam Generator | PW | 13.4 |
| 428 | Tank | Pure Steam Generator | PW | 5.2 |
| 429 | Tank | Pure Steam Generator | PW | 19.8 |
| 430 | Tank | Pure Steam Generator | PW | 1.5 |
| 431 | Tank | Pure Steam Generator | PW | 2.9 |
| 432 | Tank | Pure Steam Generator | PW | 15.9 |
| 433 | Tank | Pure Steam Generator | PW | 5.9 |
| 434 | Tank | Pure Steam Generator | PW | 3.6 |
| 435 | Tank | Pure Steam Generator | PW | 0.4 |
| 436 | Tank | Pure Steam Generator | PW | 2.8 |
| 437 | Tank | Pure Steam Generator | PW | 11.4 |
| 438 | Tank | Pure Steam Generator | PW | 5.6 |
| 439 | Tank | Pure Steam Generator | PW | 6.4 |
| 440 | Tank | Pure Steam Generator | PW | 1.4 |
| 441 | Tank | Pure Steam Generator | PW | 2.9 |
| 442 | Autoclave | Electric Steam Generator | RO | 4.8 |
| 443 | Autoclave | Electric Steam Generator | RO | 6.4 |
| 444 | Autoclave | Electric Steam Generator | RO | 6.0 |
| 445 | Autoclave | Electric Steam Generator | RO | 2.8 |
| 446 | Autoclave | Electric Steam Generator | RO | 2.8 |
| 447 | Autoclave | Electric Steam Generator | RO | 3.3 |
| 448 | Autoclave | Electric Steam Generator | RO | 1.9 |
| 449 | Autoclave | Electric Steam Generator | RO | 4.2 |
| 450 | Autoclave | Electric Steam Generator | RO | 6.8 |
| 451 | Autoclave | Electric Steam Generator | RO | 7.4 |
| 452 | Autoclave | Electric Steam Generator | RO | 1.0 |
| 453 | Autoclave | Electric Steam Generator | RO | 2.7 |
| 454 | Autoclave | Electric Steam Generator | RO | 0.5 |
| 455 | Autoclave | Electric Steam Generator | RO | 10.2 |
| 456 | Autoclave | Electric Steam Generator | RO | 2.6 |
| 457 | Autoclave | Electric Steam Generator | RO | 8.1 |
| 458 | Autoclave | Electric Steam Generator | RO | 2.3 |
| 459 | Autoclave | Electric Steam Generator | RO | 2.3 |
| 460 | Autoclave | Electric Steam Generator | RO | 1.1 |
| 461 | Autoclave | Electric Steam Generator | RO | 0.4 |
| 462 | Autoclave | Electric Steam Generator | RO | 3.2 |
| 463 | Autoclave | Electric Steam Generator | RO | 4.7 |
| 464 | Autoclave | Electric Steam Generator | RO | 2.5 |
| 465 | Autoclave | Electric Steam Generator | RO | 0.5 |
| 466 | Autoclave | Electric Steam Generator | RO | 3.3 |
| 467 | Autoclave | Electric Steam Generator | RO | 1.0 |
| 468 | Autoclave | Electric Steam Generator | RO | 6.8 |
| 469 | Autoclave | Electric Steam Generator | RO | 8.2 |
| 470 | Autoclave | Electric Steam Generator | RO | 5.3 |
| 471 | Autoclave | Electric Steam Generator | RO | 2.5 |
| 472 | Autoclave | Electric Steam Generator | RO | 2.6 |
| 473 | Autoclave | Electric Steam Generator | RO | 1.0 |
| 474 | Autoclave | Electric Steam Generator | RO | 0.1 |
| 475 | Autoclave | Electric Steam Generator | RO | 6.5 |
| 476 | Autoclave | Electric Steam Generator | RO | 7.9 |
| 477 | Autoclave | Electric Steam Generator | RO | 4.5 |
| 478 | Autoclave | Electric Steam Generator | RO | 0.5 |
| 479 | Autoclave | Electric Steam Generator | RO | 2.5 |
| 480 | Autoclave | Electric Steam Generator | RO | 1.1 |
| 481 | Steam Generator | Pure Steam Generator | RO | 0.5 |
| 482 | Steam Generator | Pure Steam Generator | RO | 0.9 |
| 483 | Steam Generator | Pure Steam Generator | RO | 3.1 |
| 484 | Autoclave | Boiler | Softener | 3.1 |
| 485 | Autoclave | Boiler | Softener | 1.2 |
| 486 | Autoclave | Boiler | Softener | 7.4 |
| 487 | Autoclave | Boiler | Softener | 2.6 |
| 488 | Autoclave | Boiler | Softener | 4.2 |
| 489 | Autoclave | Boiler | Softener | 1.9 |
| 490 | Autoclave | Electric Steam Generator | Softener | 21.3 |
| 491 | Autoclave | Boiler | Softener | 7.3 |
| 492 | Autoclave | Pure Steam Generator | Softener | 13.0 |
| 493 | Autoclave | Boiler | Softener | 3.1 |
| 494 | Autoclave | Boiler | Softener | 1.5 |
| 495 | Autoclave | Boiler | Softener | 5.2 |
| 496 | Autoclave | Boiler | Softener | 1.9 |
| 497 | Autoclave | Boiler | Softener | 0.6 |
| 498 | Autoclave | Boiler | Softener | 0.3 |
| 499 | Autoclave | Electric Steam Generator | Softener | 3.8 |
| 500 | Autoclave | Boiler | Softener | 2.0 |
| 501 | Autoclave | Pure Steam Generator | Softener | 4.1 |
| 502 | Autoclave | Boiler | Softener | 3.3 |
| 503 | Autoclave | Boiler | Softener | 0.8 |
| 504 | Autoclave | Boiler | Softener | 9.3 |
| 505 | Autoclave | Boiler | Softener | 3.1 |
| 506 | Autoclave | Boiler | Softener | 4.2 |
| 507 | Autoclave | Boiler | Softener | 2.2 |
| 508 | Autoclave | Electric Steam Generator | Softener | 9.6 |
| 509 | Autoclave | Boiler | Softener | 4.5 |
| 510 | Autoclave | Pure Steam Generator | Softener | 8.7 |
