# Supplementary material for: Analysis of water quality over non-condensable gases concentration on steam used for sterilization
Source: PLoS One. 2022 Sep 27;17(9):e0274924. doi: 10.1371/journal.pone.0274924 (PMC9514632; doi:10.1371/journal.pone.0274924)
Supplement: S2 Table — (DOCX) [file pone.0274924.s002.docx]

S2 Table. Descriptive statistics (n, mean, standard deviation, and 95% confidence intervals) of process systems were evaluated.

| **Process System** | **N** | **Mean** | **Standard Deviation** | **95% CI** |
| --- | --- | --- | --- | --- |
| Autoclave+Boiler+SW | 21 | 3.319 | 2.378 | (1.346; 5.292) |
| Autoclave+ESG+PW | 57 | 3.598 | 3.780 | (2.400; 4.796) |
| Autoclave+ ESG +RO | 39 | 3.649 | 2.643 | (2.201; 5.097) |
| Autoclave+ ESG +Softener | 03 | 11.570 | 8.910 | (6.35; 16.79) |
| Autoclave+ ESG +WFI | 78 | 1.760 | 1.213 | (0.736; 2.784) |
| Autoclave+PSG+PW | 84 | 7,813 | 8.205 | (6.826; 8.800) |
| Autoclave+PSG+ SW | 03 | 8.600 | 4.450 | (3.38; 13.82) |
| Autoclave+ PSG +WFI | 78 | 2.240 | 1.314 | (1.216; 3.264) |
| Bioreactor+ PSG +WFI | 12 | 0.883 | 1.083 | (-1.727; 3.494) |
| Filling machine+ PSG +WFI | 12 | 0.600 | 0.457 | (-2.010; 3.210) |
| Freeze dryer+ PSG +PW | 06 | 5.680 | 3.810 | (1.99; 9.38) |
| Freeze dryer+ PSG +WFI | 03 | 1.800 | 0.985 | (-3.421; 7.021) |
| Homogenizer+ PSG +WFI | 03 | 2.400 | 0.346 | (-2.821; 7.621) |
| Reactor+ PSG +PW | 12 | 5.583 | 2.507 | (2.973; 8.194) |
| Reactor+ PSG +WFI | 18 | 1.306 | 0.847 | (-0.826; 3.437) |
| Steam Generator+PSG +PW | 12 | 14.670 | 13.450 | (12.06; 17.29) |
| Steam Generator+PSG+RO | 03 | 1.500 | 1.400 | (-3.721; 6.721) |
| Steam Generator+ PSG +WFI | 09 | 3.510 | 3.380 | (0.50; 6.53) |
| Sterilization point+ PSG +PW | 18 | 6,280 | 4,700 | (4.15; 8.41) |
| Sterilization point+ PSG +WFI | 09 | 2,022 | 0,763 | (-0.992; 5.037) |
| Tank+PSG+PW | 15 | 6,61 | 5,83 | (4.27; 8.94) |
| Tank+PSG+WFI | 15 | 0,893 | 0,859 | (-1.442; 3.228) |

PSG = Pure Steam Generator; ESG = Electric Steam Generator; PW = purified water; SW = softened water; RO = reverse osmosis water; WFI = water for injection.
