# Supplementary figures and images for: Analysis of water quality over non-condensable gases concentration on steam used for sterilization
Source: PLoS One. 2022 Sep 27;17(9):e0274924. doi: 10.1371/journal.pone.0274924 (PMC9514632; doi:10.1371/journal.pone.0274924)

S3 Figures. NCGs Histogram for process systems (H0 and 95% confidence interval for the mean)


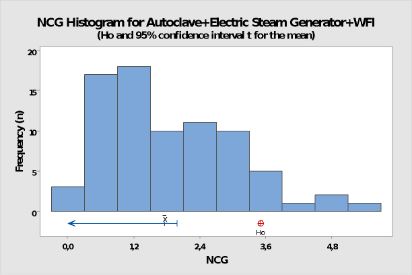


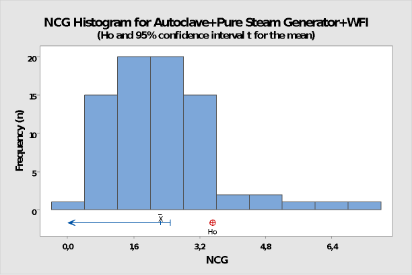


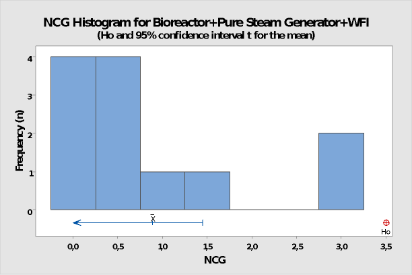


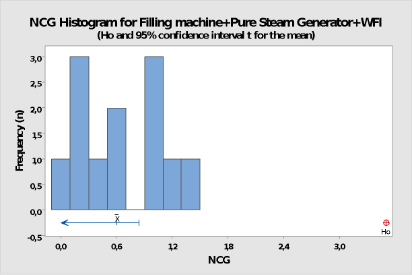


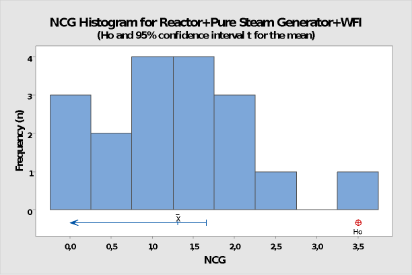


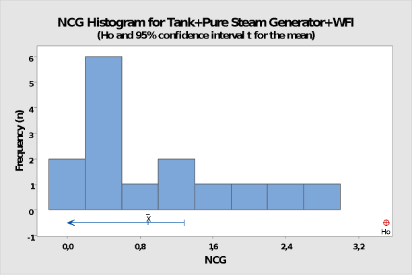

Supplement: S1 Fig — (DOCX) [file pone.0274924.s003.docx]
